# Down-Regulation of the Proteoglycan Decorin Fills in the Tumor-Promoting Phenotype of Ionizing Radiation-Induced Senescent Human Breast Stromal Fibroblasts

**DOI:** 10.3390/cancers13081987

**Published:** 2021-04-20

**Authors:** Eleni Mavrogonatou, Adamantia Papadopoulou, Asimina Fotopoulou, Stathis Tsimelis, Heba Bassiony, Andreas M. Yiacoumettis, Petros N. Panagiotou, Harris Pratsinis, Dimitris Kletsas

**Affiliations:** 1 Laboratory of Cell Proliferation and Ageing, Institute of Biosciences and Applications, National Centre for Scientific Research “Demokritos”, 15341 Athens, Greece; elmavro@bio.demokritos.gr (E.M.); apapad@bio.demokritos.gr (A.P.); asiminaf@bio.demokritos.gr (A.F.); eftsimel@bio.demokritos.gr (S.T.); hprats@bio.demokritos.gr (H.P.); 2 Department of Zoology, Faculty of Science, Cairo University, 12613 Giza, Egypt; heba_b683@hotmail.com; 3 Plastic and Reconstructive Surgery Department, Metropolitan General Hospital, 15562 Athens, Greece; yiacoume@gmail.com; 4 Department of Plastic Surgery and Burns Unit, KAT General Hospital of Athens, 14561 Athens, Greece; p-pan@otenet.gr

**Keywords:** human breast stromal fibroblasts, senescence, γ-irradiation, decorin, breast cancer cells, growth factors, bFGF, VEGF, autophagy

## Abstract

**Simple Summary:**

Ionizing radiation (a typical remedy for breast cancer) results in the premature senescence of the adjacent to the neoplastic cells stromal fibroblasts. Here, we showed that these senescent fibroblasts are characterized by the down-regulation of the small leucine-rich proteoglycan decorin, a poor prognostic factor for the progression of the disease. Decorin down-regulation is mediated by secreted growth factors in an autocrine and paracrine (due to the interaction with breast cancer cells) manner, with bFGF and VEGF being the key players of this regulation in young and senescent breast stromal fibroblasts. Autophagy activation increases decorin mRNA levels, indicating that impaired autophagy is implicated in the reduction in decorin in this cell model. Decorin down-regulation acts additively to the already tumor-promoting phenotype of ionizing radiation-induced prematurely senescent human stromal fibroblasts, confirming that stromal senescence is a side-effect of radiotherapy that should be taken into account in the design of anticancer treatments.

**Abstract:**

Down-regulation of the small leucine-rich proteoglycan decorin in the stroma is considered a poor prognostic factor for breast cancer progression. Ionizing radiation, an established treatment for breast cancer, provokes the premature senescence of the adjacent to the tumor stromal fibroblasts. Here, we showed that senescent human breast stromal fibroblasts are characterized by the down-regulation of decorin at the mRNA and protein level, as well as by its decreased deposition in the pericellular extracellular matrix in vitro. Senescence-associated decorin down-regulation is a long-lasting process rather than an immediate response to γ-irradiation. Growth factors were demonstrated to participate in an autocrine manner in decorin down-regulation, with bFGF and VEGF being the critical mediators of the phenomenon. Autophagy inhibition by chloroquine reduced decorin mRNA levels, while autophagy activation using the mTOR inhibitor rapamycin enhanced decorin transcription. Interestingly, the secretome from a series of both untreated and irradiated human breast cancer cell lines with different molecular profiles inhibited decorin expression in young and senescent stromal fibroblasts, which was annulled by SU5402, a bFGF and VEGF inhibitor. The novel phenotypic trait of senescent human breast stromal fibroblasts revealed here is added to their already described cancer-promoting role via the formation of a tumor-permissive environment.

## 1. Introduction

Cancer is one of the major causes of morbidity and mortality worldwide [1]. Among the numerous different types of cancer, breast cancer is generally regarded as the most common malignancy, predominantly in female patients, and the second leading cause of cancer-related deaths in women around the world [1,2]. Among generally used remedies for breast cancer, including hormonal therapy, chemotherapy and surgery, radiotherapy remains a standard arm in the arsenal of oncologists in order to cope with this great challenge to women’s health, aiming at improving the prognosis of the disease [1,3].

For many years carcinogenesis and tumor expansion were solely attributed to the genetic and molecular alterations obtained by neoplastic cells, underappreciating the critical role of the stromal components in the process of cancer development [4,5]. The stroma embracing cancer cells constitutes a high fraction of the solid tumor’s volume. It mainly consists of extracellular matrix (ECM) that encompasses the stromal cells, including fibroblasts, endothelial cells, specialized mesenchymal cells and immune cells [4]. Fibroblasts represent the most important stromal cell type, as they are the main producers of the ECM components (e.g., collagens and proteoglycans) and ECM-degrading enzymes, as well as of the soluble mediators—such as growth factors and chemokines—that shape tissue structure and regulate cell and tissue functions, delineating the composition and architecture of the tumor surrounding milieu [4,6]. By now, the tumor microenvironment has emerged as a key participant in cancer progression via the modified synthesis and deposition of several ECM constituents and via its complex interactions with malignant cells [4,5,7,8,9]. Indeed, a great number of studies have suggested that whereas a normal microenvironment can impede tumor growth, an activated stroma can be an accomplice in cancer development [10,11,12,13,14,15].

Ionizing radiation, which is commonly used to treat breast cancer, inevitably affects stromal cells located within or around tumors and it can render them senescent [16,17,18,19]. Cellular senescence is a state of an irreversible cell cycle arrest, first observed in human embryonic lung fibroblasts [20], but yet known to be met by the majority of cell types. There are two kinds of cellular senescence: the “replicative senescence” attributed to telomere attrition arising from the consecutive replications of the cells and the “stress-induced premature senescence” (SIPS) manifested as the result of several genotoxic stresses encountered by the cells [21,22,23]. Irrespective of their origin, all senescent cells are characterized by a cease in their proliferation connected with the up-regulation of several cell cycle regulators, i.e., p21^WAF1^ and p16^INK4a^. Beyond their inability to proliferate and their distinct morphological traits (e.g., enlarged and irregular shape, accumulation of the highly oxidized lysosomal byproduct lipofuscin), senescent cells possess a catabolic and pro-inflammatory phenotype, namely the “senescence-associated secretory phenotype” (SASP) (consisting of soluble inflammatory mediators, proteolytic enzymes or growth factors and insoluble ECM components) [4,5,24]. SASP factors could be responsible for tissue remodeling towards the creation of a permissive for tumor growth microenvironment [18,25,26]. Nevertheless, while the expression and function of soluble SASP factors have been extensively studied, less attention has been devoted to insoluble SASP factors, e.g., ECM components.

We have recently shown that ionizing radiation results in the accumulation of prematurely senescent breast stromal fibroblasts both in vitro and in vivo [19]. Ionizing radiation-induced senescent breast stromal fibroblasts have been shown to be characterized by an enhanced expression and enzymatic activity of ECM-degrading proteases (e.g., matrix mettaloproteases, MMPs) in parallel to the down-regulation and decreased biosynthesis of type I collagen. This catabolic phenotype is complemented by the overexpression of the cell surface heparan sulfate proteoglycan syndecan-1, a poor prognostic factor for breast cancer development that was found to be regulated by transforming growth factor-β (TGF-β) through the Smad pathway and the transcription factor Sp1 in an autocrine and paracrine manner.

Proteoglycans may exert distinct and often opposite functions during cancer pathogenesis due to their high complexity, heterogeneity and disparate structure and ability to interact with both ligands and receptors [27]. The small leucine-rich proteoglycan (SLRP) decorin, for example, in contrast to syndecan-1 is known to have an antitumorigenic effect when overexpressed in the stroma [28,29,30]. Decorin is the archetypical and most extensively studied representative of the 18-member SLRP family [31,32,33] that owns a single, covalently attached N-terminal glycosaminoglycan (GAG) chain consisting of dermatan or chondroitin sulfate, a protein core of twelve leucine-rich tandem repeats, and a class-specific C-terminal Ear domain [34]. Decorin is a stromal proteoglycan primarily synthesized by fibroblasts, vascular endothelial cells and smooth muscle cells and has numerous binding partners, including ECM components, such as collagens, and growth factors, i.e., vascular endothelial growth factor (VEGF), TGF-β, platelet derived growth factor (PDGF), fibroblast growth factor (FGF), insulin-like growth factor (IGF), connective tissue growth factor (CTGF) and hepatocyte growth factor (HGF) [35,36]. Within the tumor microenvironment, decorin directly engages and down-regulates multiple receptor tyrosine kinases including the epidermal growth factor receptor (EGFR), the HGF receptor Met, the IGF-I receptor (IGFIR), the VEGF receptor (VEGFR) and the PDGF receptor (PDGFR), often overexpressed in cancer cells [31,35,36]. Recently, decorin has been established as an autophagic signal transducer by initiating endothelial cell autophagy and by inducing tumor cell mitophagy [37,38,39]. Overall, decorin constitutes a potent antitumorigenic signal by suppressing tumor cell proliferation, migration, invasion, metastasis and angiogenesis, rendering high stromal decorin expression a significant predictive marker of higher survival rates and the good prognosis in patients with breast cancer, according to a comprehensive and detailed meta-analysis that evaluated the prognostic value of stromal decorin expression [3,40].

Having in mind the accumulation of senescent fibroblasts in irradiated breast stroma [19] and given that expression levels of decorin in the stroma could serve as a prognostic biomarker for breast cancer progression, here we studied the expression profile of decorin in ionizing radiation-induced senescent human breast stromal fibroblasts. Furthermore, we investigated the mechanisms underlying the regulation of decorin expression in breast stromal fibroblasts and the cellular processes and soluble factors participating in this regulation. Finally, we assessed the implication of paracrine interactions with human breast cancer cells on the observed phenomena.

## 2. Materials and Methods

### 2.1. Reagents and Chemicals

PDGF-BB and human recombinant transforming growth factor-beta 1 (TGF-β1) were purchased from Biochrom GmbH (Berlin, Germany) and were used at concentrations of 10 and 5 ng/mL, respectively; basic FGF (bFGF) and IGF-I were obtained from R&D Systems Europe (Abingdon, UK) and were applied at 10 and 100 ng/mL, respectively; human vascular endothelial growth factor (VEGF)-165 was purchased by Cell Signaling Technology (Beverly, MA, USA) and was used at 10, 20 and 50 ng/mL; epidermal growth factor (EGF) used at a concentration of 100 ng/mL was supplied by Sigma (St. Louis, MO, USA). The PDGFR kinase inhibitor STI571 (2 μΜ) was purchased from Novartis AG (Basel, Switzerland); the VEGFR and FGFR inhibitor SU5402 (20 μM), the EGFR kinase inhibitor tyrphostin AG1478 (3 μM) and the IGFIR kinase inhibitor tyrphostin I-OMe-AG538 (12 μM) were obtained from Calbiochem-Merck KGaA (Darmstadt, Germany); the TGF-β type I receptor (TGFβR1) kinase inhibitor SB431542 (10 μΜ) and the VEGFR inhibitor SU5416 (1 μΜ) were from Sigma, while the FGFR inhibitor AZD4547 (21 nM) was purchased from MedChemExpress (Monmouth Junction, NJ, USA). The mammalian target of rapamycin (mTOR) inhibitor rapamycin (500 nM) and the autophagy inhibitor chloroquine (30 μΜ) were supplied by Sigma.

### 2.2. Cells, Cell Culture Conditions, Exposure to γ-Irradiation and Induction of Premature Senescence

Human breast stromal fibroblasts used in this study were from a pre-existing cell bank of the Laboratory of Cell Proliferation and Ageing [19]. In brief, primary cell cultures were established after dissection and collagenase digestion of normal tissues from consenting volunteers undergoing surgery under the approval of the Bioethics Committee of the NCSR “Demokritos”. Human breast cancer epithelial cells MCF-7, ZR-75-1 and MDA-MB-231 were purchased from the American Type Culture Collection (ATCC, Rockville, MD, USA), while the human mammary gland adenocarcinoma cell lines SKBR3 and MDA-MB-468 were generously provided by Prof. Martin Götte and Dr. Vassilis Zoumpourlis, respectively. All cells were routinely cultured in DMEM supplemented with penicillin (100 U/mL)/streptomycin (100 mg/mL) (obtained from Biosera, Nuaille, France) and 10% (*v*/*v*) FBS (from Gibco BRL, Invitrogen, Paisley, UK), were maintained in a humidified atmosphere of 5% CO_2_ at 37 °C and were subcultured when confluent using a trypsin/citrate (0.25%:0.30% *w*/*v*) solution.

For the induction of premature senescence, primary human breast stromal fibroblasts were exposed to a cumulative dose of 50 Gy of γ-irradiation followed by serial subculturing, as previously reported [18,19,41]. Exposure of cells to γ-irradiation was performed in a ^60^Co gamma source (Gamma Chamber 4000 A, Isotope Group, Bhadha Atomic Research Company, Trombay, Bombay, India) at a rate of 2.5 Gy/min. To assess the immediate effect of ionizing radiation on breast stromal fibroblasts and breast cancer cells, cells were exposed to 4 or 10 Gy of γ-irradiation.

### 2.3. Senescence-Associated β-Galactosidase (SA-β-Gal) Staining

SA-β-Gal staining was performed as previously described [42]. In brief, cells were grown on glass coverslips before fixation with 3% (*v*/*v*) formaldehyde in PBS. Samples were then stained with SA-β-Gal staining solution (40 mM citric acid/sodium phosphate pH 6.0, 150 mM NaCl and 2 mM MgCl_2_ containing 5 mM potassium ferricyanide, 5 mM potassium ferrocyanide and 1 mg/mL X-Gal) and incubated at 37 °C. SA-β-Gal-positive or -negative cells were observed under a Zeiss Axioplan 2 phase contrast microscope (Carl Zeiss, Jena, Germany).

### 2.4. SenTraGor Immunofluorescence Staining

For the immunofluorescence staining of the lipofuscin-containing senescent cells [43] with SenTraGor (Lab Supplies Scientific, Athens, Greece), human primary breast stromal fibroblasts were plated on glass coverslips and fixed with 4% (*v*/*v*) formaldehyde in PBS. Staining was performed according to the manufacturer’s instructions. In brief, coverslips were washed with TBS, followed by a wash with 50% (*v*/*v*) ethanol and a wash with 70% (*v*/*v*) ethanol. Samples were then incubated with SenTraGor reagent (the chemical compound GL13 linked with biotin) until detection of the signal under a light microscope. After removal of the excess SenTraGor reagent, samples were washed with 50% (*v*/*v*) ethanol and TBS, before incubation with a phycoerythrin-conjugated anti-biotin antibody (Invitrogen) and counterstained with 2 μg/mL 4′,6-diamino-2-phenylindole (DAPI) dihydrochloride (Sigma). Samples were visualized under a confocal laser scanning microscope (TCS SP8 multiphoton confocal microscope, Leica, Mannheim, Germany).

### 2.5. Estimation of Cell Proliferation by 5-Bromo-2′-Deoxyuridine (BrdU) Incorporation

For the estimation of cell proliferation, cells were allowed to attach on glass coverslips before labeling with 50 μM BrdU for 48 h, as reported before [19,41,42]. Cells were then fixed with 4% (*v*/*v*) formaldehyde in PBS for 10 min, permeabilized with 0.2% (*v*/*v*) Triton X-100 in PBS for 10 min and denaturation of DNA was achieved after treatment with 2 N HCl for 30 min. After blocking with 0.5% (*v*/*v*) gelatin in PBS, samples were incubated with an anti-BrdU-FITC antibody purchased from BioLegend (SanDiego, CA, USA) at 4 °C and counterstained with 2 μg/mL DAPI in PBS for 20 min. The percentage of cells with a proliferative potential in a given cell population was calculated by dividing the number of BrdU-positive nuclei by the number of the DAPI-positive nuclei, as counted under a Zeiss Axioplan 2 fluorescent microscope.

### 2.6. Total Protein Extraction, Preparation of Human Breast Stromal Fibroblast-Derived Extracellular Matrix (ECM) and Western Blot Analysis

Protein samples for SDS electrophoresis were collected as previously described in a Laemmli sample buffer containing a protease-inhibitor cocktail (Sigma) [44]. Primary cultures of human breast stromal fibroblasts were used in order to obtain fibroblast-derived ECM. Briefly, young fibroblasts, as well as ionizing radiation-mediated premature senescent ones, were plated into 60-mm culture dishes. Cells were cultured in DMEM supplemented with 10% (*v*/*v*) FBS for 15 days in order to deposit a sufficient amount of ECM, with medium change every two days along with 50 μg/mL of L-ascorbic acid phosphate (Sigma) from the 8th day of culture. Then, in order to prepare decellularized ECM, the cultures were washed once with PBS at room temperature and were incubated for 10 min at 4 °C and under mild agitation with sodium deoxycholate solution (0.5% (*w*/*v*) sodium deoxycholate, 0.5 M Tris-HCl, pH 8.0), followed by a wash with PBS (thrice for 5 min at 4 °C) [45]. Finally, decellularized matrices were harvested from the culture dishes by using hot 2X Laemmli sample buffer supplemented with a protease-inhibitor cocktail (Sigma).

Total cellular protein and ECM extracts were separated using 7.5% (*w*/*v*) polyacrylamide gels and proteins were transferred onto Amersham Hybond PVDF membranes (GE Healthcare, Buckinghamshire, UK). Western blot analysis was performed as reported before [19,44] using a human decorin antibody (R&D Systems Europe). Equal loading for protein extracts was confirmed by Western blot analysis using an antibody against human glyceraldehyde 3-phosphate dehydrogenase (GAPDH) (Santa Cruz Biotechnology, Santa Cruz, CA, USA), while appropriate quantities of ECM extracts were loaded on the gels based on the number of the respective ECM-producing cells.

### 2.7. Preparation of Cancer Cell Cultures’ Conditioned Media

Conditioned media from breast cancer cells were collected as described previously [19] with slight modifications. Briefly, after three washes with serum-free DMEM, cells grown in 100-mm culture dishes were incubated with serum-free DMEM for 3 h. Cell cultures were then exposed or not to 4 and 10 Gy of γ-irradiation and further incubated for another 24 h. Conditioned media were collected and centrifuged (500 g/15 min) for the removal of cell debris, aliquoted and stored at −80 °C until use. Human stromal fibroblasts were exposed to media conditioned by breast cancer cells (50% (*v*/*v*)).

### 2.8. Flow Cytometric Analysis

The effect of γ-irradiation on the cell cycle progression of breast cancer epithelial cells was assessed using flow cytometry, as previously described [44,46]. Cells were exposed to 4 and 10 Gy of ionizing radiation and 24 h later they were harvested by trypsinization, washed with PBS, fixed with 50% (*v*/*v*) ice-cold ethanol and stored at 4 °C until further analysis. Samples were stained with a solution containing 50 μg/mL propidium iodide (PI), 5 mM MgCl_2_ and 10 μg/mL RNase A. Cell cycle analysis was performed in a FACS Calibur flow cytometer using the Cell Quest software (Becton Dickinson, San Jose, CA, USA). Data analysis was performed using Modfit (Verity Software House, Topsham, ME, USA) and FCS Express version 7 (De Novo Software, Los Angeles, CA, USA).

### 2.9. RNA Extraction and Reverse Transcription (RT)-Quantitative (q)PCR

RNA was extracted with Trizol (Invitrogen) according to the manufacturer’s instructions, as described before [19,41,42,47] and first-strand cDNA synthesis was performed with the PrimeScript RT Reagent Kit (Takara, Tokyo, Japan) in 10-μL reactions using 300–500 ng RNA as template. Quantitative PCR experiments were performed in 20-μL reactions with the qPCRBIO SyGreen Mix Lo-ROX (PCR Biosystems Ltd., London, UK) in a MX3000P cycler (Stratagene, La Jolla, CA, USA). Relative mRNA expression was calculated using the 2^−ΔΔCt^ method [48], as reported previously [19,41,42,47]. Mean Ct values of the selected genes were normalized to that of GAPDH which served as the reference gene. Sequences of the primers used in this study are presented in Table 1.

### 2.10. Statistical Analysis

Data presented in the graphs are mean values ± standard deviations from at least three independent experiments in cells deriving from no less than two donors. Differences were considered statistically significant when *p* < 0.05 (Student’s *t* test).

## 3. Results

### 3.1. Ionizing Radiation-Induced Prematurely Senescent Human Breast Stromal Fibroblasts Are Characterized by Decreased Decorin Expression

**Figure 1:**
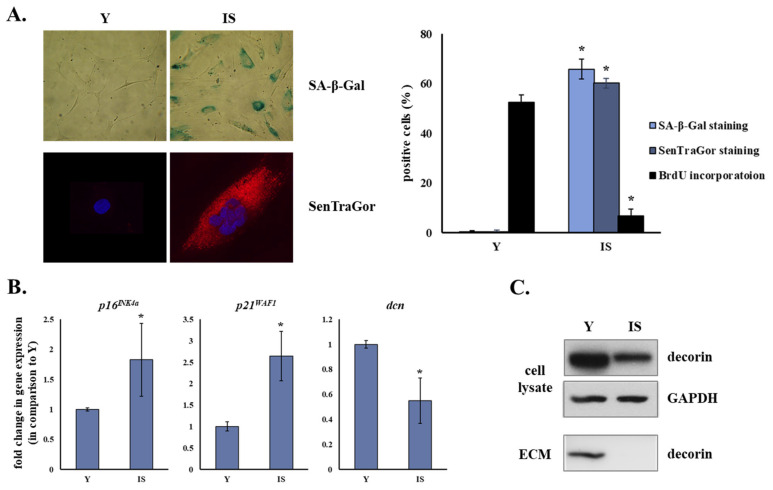
Ionizing radiation leads to the premature senescence of human breast stromal fibroblasts, characterized by the down-regulation of the small leucine-rich proteoglycan decorin. Young (Y) and ionizing radiation-induced senescent (IS) human breast stromal fibroblasts were grown in DMEM containing 10% (*v*/*v*) FBS before fixation and staining for β galactosidase activity, lipofuscin accumulation and BrdU incorporation (**A**), RNA extraction and gene expression analysis (**B**) and protein or extracellular matrix (ECM) extraction and Western blot analysis (**C**). Glyceraldehyde-3-phosphate dehydrogenase (GAPDH) served as the reference gene in the RT-qPCR analysis and as the loading control in the Western blot analysis of cell lysates. ECM extracts were loaded based on equal cell number of the ECM-producing cells. In the graphs, means ± standard deviations from three and four independent experiments for (**A**,**B**), respectively, are depicted. Asterisks (*) represent statistically significant differences in comparison to Y (Student’s *t* test, *p* < 0.05). SA-β-Gal staining, SenTraGor staining and Western blot analyses for decorin were repeated three times and a representative experiment is presented here. dcn: decorin.

Human breast stromal fibroblasts exposed to γ-irradiation were driven to premature senescence, as confirmed by their inadequacy for novel DNA synthesis and their higher percentage of positive SA-β-Gal staining (Figure 1A). BrdU incorporation declined from 52.6 ± 2.8% in early passage cells (henceforth cited as “young” cells) to 6.8 ± 2.8% in ionizing radiation-induced senescent cells (*p* < 0.05) in parallel to the rise of the positive SA-β-Gal staining percentage from 0.3 ± 0.5% to 65.9 ± 4.1% (*p* < 0.05) in young and senescent cells, respectively. Furthermore, these prematurely senescent cells were characterized by lipofuscin accumulation denoted by the positive SenTraGor staining (0.4 ± 0.6% in young cells and 60.3 ± 1.9% in senescent cells (*p* < 0.05)) (Figure 1A).

In addition to the up-regulation of the known cyclin-dependent kinase inhibitors and hallmarks of senescence p16^INK4a^ and p21^WAF1^, ionizing radiation-induced senescent human breast stromal fibroblasts showed decreased decorin mRNA levels in comparison to young breast stromal fibroblasts (Figure 1B). Even more, decorin was found to be down-regulated in ionizing radiation-induced senescent human breast stromal fibroblasts at the protein level (Figure 1C and Supplementary Figure S1).

Beyond the detection of the intracellular concentration of decorin, we also checked for its incorporation in the pericellular ECM. Accordingly, we prepared autologous extracellular matrices from cultures of young and senescent human breast stromal fibroblasts, after cell removal by sodium deoxycholate extraction. As shown in Figure 1C and Supplementary Figure S1, while decorin was observed in “young” matrices, it was undetectable in “senescent” ones. This indicates a significant decrease in the production of decorin by senescent cells, as well as in its accumulation in the pericellular space.

### 3.2. Decorin Down-Regulation in Senescent Human Breast Stromal Fibroblasts Is a Long-Term Supervention Rather Than an Immediate Response of the Cells to the Genotoxic Effect of Ionizing Radiation

**Figure 2:**
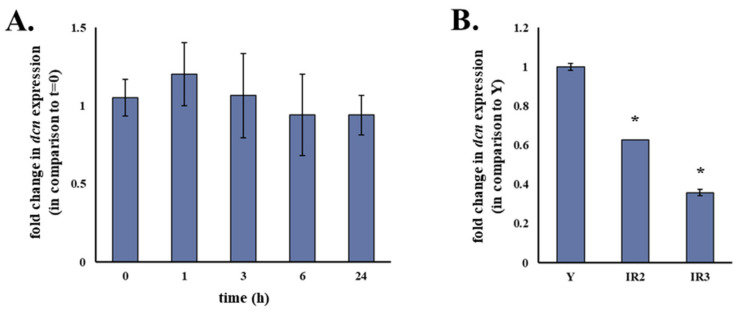
Decorin down-regulation in ionizing radiation-induced senescent human breast stromal fibroblasts seems to be a long-term response of the cells rather than an immediate effect of γ-irradiation. Confluent human breast stromal fibroblasts’ cultures in DMEM supplemented with 10% (*v*/*v*) FBS were exposed to 4 Gy of γ-irradiation and further incubated at 37 °C for the designated time-points (**A**) or were exposed to 50 Gy of γ-irradiation and subjected to subculturing thrice (**B**) before RNA extraction. mRNA levels of decorin (dcn) were measured by real-time PCR following first-strand cDNA synthesis. Mean Ct values of dcn in each sample were normalized to that of glyceraldehyde-3-phosphate dehydrogenase (GAPDH). Ratios of the expression levels of treated cells to those of the respective untreated control are presented as mean values ± standard deviations of three independent experiments. Statistically significant differences (Student’s *t* test, *p* < 0.05) from Y are marked by asterisks (*). IR2: second subculture post-irradiation; IR3: third subculture post-irradiation.

To elucidate whether reduced decorin expression in ionizing radiation-induced senescent human stromal fibroblasts was a response of the cells to the immediate genotoxic insult of γ-irradiation or a senescence-associated trait, we exposed the cells to 4 Gy of ionizing radiation (a typical dose of a therapeutic radiotherapy session) and analyzed the transcriptional regulation of decorin in different time-points (extending from 1 to 24 h post-irradiation). As shown in Figure 2A, no change in decorin gene expression was observed 1, 3, 6 or even 24 h after the cells were exposed to γ-irradiation. On the contrary, it seems that decorin down-regulation is a long-lasting process which is exacerbated in the course of the establishment of the senescent phenotype (Figure 2B).

### 3.3. Decorin mRNA Levels Are Decreased in Human Breast Stromal Fibroblasts as a Response to Exogenously Supplied Growth Factors

**Figure 3:**
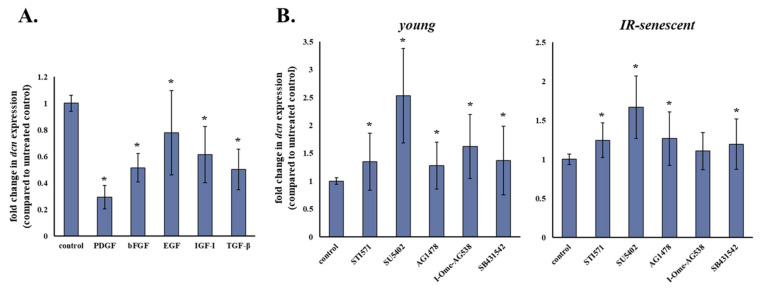
Decorin expression is decreased in human breast stromal fibroblasts as a response to exogenous growth factors. (**A**) Human breast stromal fibroblasts were subjected to starvation by medium change to serum-free DMEM for 24 h and were then treated with PDGF (10 ng/mL), bFGF (10 ng/mL), EGF (100 ng/mL), IGF-I (100 ng/mL) or TGF-β (5 ng/mL) for another 24 h before RNA extraction and RT-qPCR analysis. (**B**) Young and ionizing radiation-induced senescent human breast stromal fibroblasts were incubated for 24 h with the growth factor receptor inhibitors (STI571 for PDGFR (2 μΜ); SU5402 for FGFR and VEGFR (20 μΜ); AG1478 for EGFR (3 μΜ); I-OMe-AG538 for IGFIR (12 μΜ); SB431542 for TGFβR1 (10 μΜ)). Then, RNA was extracted and decorin (dcn) expression was estimated by real-time PCR after first-strand cDNA synthesis. Glyceraldehyde-3-phosphate dehydrogenase (GAPDH) was used as the reference gene. Ratios of the expression levels of treated cells to those of the untreated control are shown as means ± standard deviations of eight and five independent experiments for (**A**,**B**), respectively. Asterisks (*) stand for statistically significant differences in comparison to the respective untreated control (Student’s *t* test, *p* < 0.05). IR: ionizing radiation.

Given the reported paracrine effect of soluble factors secreted by breast cancer cells on breast stromal fibroblasts [19]—we assessed the effect of selected exogenous and paracrine growth factors (i.e., PDGF, bFGF, EGF, IGF-I and TGF-β) on the transcriptional regulation of decorin. As demonstrated in Figure 3A, all growth factors tested led to a statistically significant down-regulation of decorin in human breast stromal fibroblasts.

To further investigate the role of the afore-mentioned growth factors in decorin gene expression, we treated both young and senescent human breast stromal fibroblasts with specific pharmacological inhibitors of the growth factors’ receptor kinases (STI571 for PDGFR, SU5402 for FGFR and VEGFR, AG1478 for EGFR, I-OMe-AG538 for IGFIR and SB431542 for TGFβR1). Concentrations of the inhibitors STI571, SU5402, AG1478, I-OMe-AG538 and SB431542 have been selected in the past based on their ability to completely block the response of breast stromal fibroblasts to PDGF, bFGF, EGF, IGF-I, and TGF-β, respectively, while not interfering with the response to other growth factors, and are similar to the ones used in our previously published work [19]. In agreement with the inhibitory effect of growth factors on decorin expression, all their respective receptors’ inhibitors enhanced decorin expression, indicating an autocrine action. Of note, the most potent effect was observed in cells treated with SU5402, which resulted in the highest statistically significant increase in decorin expression amongst all inhibitors used in young, as well as in senescent human stromal fibroblasts (Figure 3B).

### 3.4. bFGF and VEGF Seem to Be the Main Negative Regulators of Decorin Expression in Human Breast Stromal Fibroblasts

**Figure 4:**
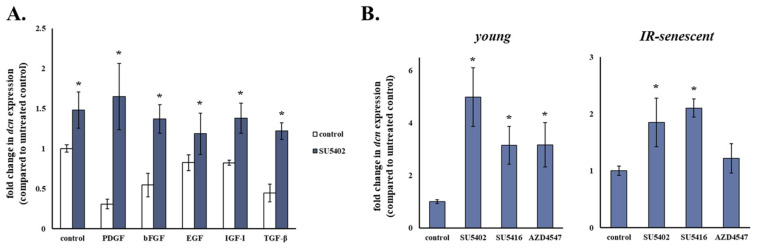
bFGF and VEGF seem to be the key growth factors down-regulating decorin expression in human stromal fibroblasts. (**A**) Human breast stromal fibroblasts were serum starved for 24 h, pre-incubated with 20 μΜ of SU5402 for 1 h and then treated with PDGF (10 ng/mL), bFGF (10 ng/mL), EGF (100 ng/mL), IGF-I (100 ng/mL) or TGF-β (5 ng/mL) for another 24 h before RNA extraction, first-strand cDNA synthesis and real-time PCR analysis. (**B**) Young and ionizing radiation-induced senescent human breast stromal fibroblasts were incubated for 24 h with the designated growth factor receptor inhibitors (SU5402 for FGFR and VEGFR (20 μΜ); SU5416 for VEGFR (1 μΜ); AZD4547 for FGFR (21 nΜ)). RNA was extracted and decorin (dcn) expression was estimated by RT-qPCR with glyceraldehyde-3-phosphate dehydrogenase (GAPDH) serving as the reference gene. Relative mRNA levels compared to the respective untreated control are presented as means ± standard deviations of three experiments. Statistically significant differences in comparison to the respective control without the inhibitor are marked by asterisks (*) (Student’s *t* test, *p* < 0.05). IR: ionizing radiation.

Taking into account the potency of SU5402 to increase decorin mRNA levels of human breast stromal fibroblasts shown earlier, we then assessed whether treatment with this particular inhibitor would result in the abolishment of the growth factor-induced decorin down-regulation in these cells. We found that SU5402 led to the abrogation of decorin down-regulation induced by all growth factors tested (Figure 4A), thus corroborating the key role of bFGF and VEGF in modulating decorin expression in human breast stromal fibroblasts and suggesting that the action of PDGF, EGF, IGF-I and TGF-β is also mediated by these two growth factors, most probably secondarily via an autocrine loop.

Since SU5402 is known to block both VEGFR and FGFR, subsequently we used two other pharmacological inhibitors, SU5416 and AZD4547, specific for VEGFR and FGFR, respectively, in an attempt to decouple the role of these particular growth factors in the observed phenomenon. As shown in Figure 4B, negative regulation of decorin seems to be attributed equally to VEGF and bFGF in young human breast stromal fibroblasts, as incubation with all SU5402, SU5416 and AZD4547 increased decorin mRNA levels in comparison to the untreated control. On the other hand, in senescent human breast stromal fibroblasts negative regulation of decorin expression appears to depend solely on VEGF, as AZD4547 had no statistically significant effect, at the same time that SU5402 and SU5416 displayed a similar action (Figure 4B).

### 3.5. VEGF Seems to Participate in Decorin Down-Regulation as Part of Its Intracrine Functions in Human Breast Stromal Fibroblasts

**Figure 5:**
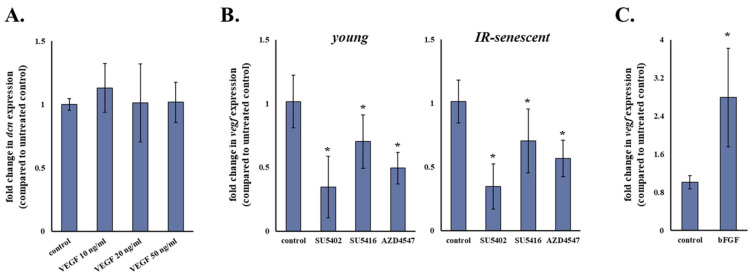
VEGF participation in the regulation of decorin expression in human breast stromal fibroblasts. Human breast stromal fibroblasts were serum starved for 24 h and then treated with VEGF (10, 20 and 50 ng/mL) (**A**), were exposed to 20 μΜ of the FGFR and VEGFR inhibitor SU5402, 1 μΜ of the VEGFR inhibitor SU5416 and 21 nM of the FGFR inhibitor AZD4547 (**B**) or were serum starved for 24 h and then treated with bFGF (10 ng/mL) (**C**) for 24 h before RNA extraction, first-strand cDNA synthesis and real-time PCR analysis with glyceraldehyde-3-phosphate dehydrogenase (GAPDH) serving as the reference gene. Relative mRNA levels compared to the respective untreated control are presented as means ± standard deviations of three experiments. Statistically significant differences in comparison to the respective control without the inhibitor are designated by asterisks (*) (Student’s *t* test, *p* < 0.05). IR: ionizing radiation.

Since treatment with the specific VEGFR inhibitor SU5416 led to the induction of decorin expression in human breast stromal fibroblasts revealing the participation of VEGF, we then assessed the effect of exogenously supplied VEGF on decorin expression (Figure 5A). Interestingly, treatment with three different VEGF concentrations (10, 20 and 50 ng/mL) resulted in no statistically significant alteration of decorin mRNA levels in human breast stromal fibroblasts. On the other hand, FGFR and/or VEGFR inhibition decreased mRNA levels of VEGF itself (Figure 5B), suggesting an implication of the particular growth factor in the regulation of decorin expression by acting in an intracrine manner.

Down-regulation of VEGF expression by the FGFR inhibitors SU5402 and AZD4547 implied that VEGF is controlled by bFGF and for that reason we investigated the effect of exogenous bFGF on VEGF expression in human breast stromal fibroblasts. As shown in Figure 5C, bFGF administration to human breast stromal fibroblasts induced VEGF overexpression. All the above indicate that bFGF regulates decorin expression—at least in part—via VEGF.

### 3.6. Autophagy Is Implicated in the Regulation of Decorin Expression in Human Breast Stromal Fibroblasts

**Figure 6:**
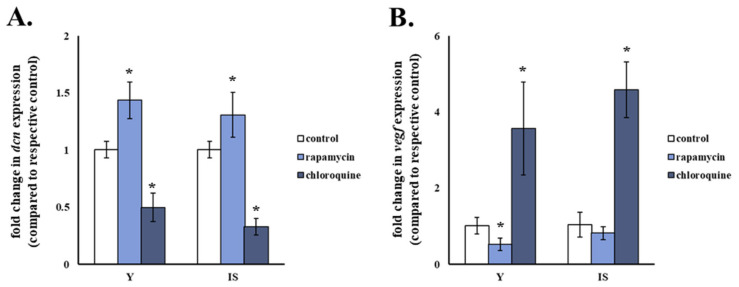
Decorin expression in human breast stromal fibroblasts is autophagy-induced. (**A**) Young (Y) and ionizing radiation-induced senescent (IS) human breast stromal fibroblasts were treated with 500 nM of the autophagy activator rapamycin or 30 μM of the autophagy inhibitor chloroquine for 24 h before RNA extraction and RT-qPCR analysis for decorin (dcn) expression (**A**) or VEGF expression (**B**). Glyceraldehyde-3-phosphate dehydrogenase (GAPDH) was used as the reference gene. Ratios of gene expression levels of treated cells to those of the respective untreated control are shown as mean values ± standard deviations of four and three independent experiments for (**A**,**B**), respectively. Asterisks (*) pinpoint statistically significant differences in comparison to the respective untreated control (Student’s *t* test, *p* < 0.05).

It has been reported previously that activation of autophagy leads to decorin overexpression in NIH-3T3 fibroblasts [49]. Based on this finding, we investigated the role of a functional autophagic flux in the regulation of decorin expression in human breast stromal fibroblasts. The autophagy activator rapamycin and the autophagy inhibitor chloroquine resulted in the statistically significant increase and decrease in decorin mRNA levels, respectively, in both young and senescent human breast stromal fibroblasts in comparison to the respective untreated control (Figure 6A). Moreover, activation of autophagy by rapamycin decreased VEGF mRNA levels, while inhibition of autophagy by chloroquine led to the up-regulation of VEGF in young and senescent human breast stromal fibroblasts (Figure 6B).

### 3.7. Breast Cancer Cells Stimulate the Down-Regulation of Decorin in Young and Senescent Human Breast Stromal Fibroblasts in a Paracrine Manner

**Figure 7:**
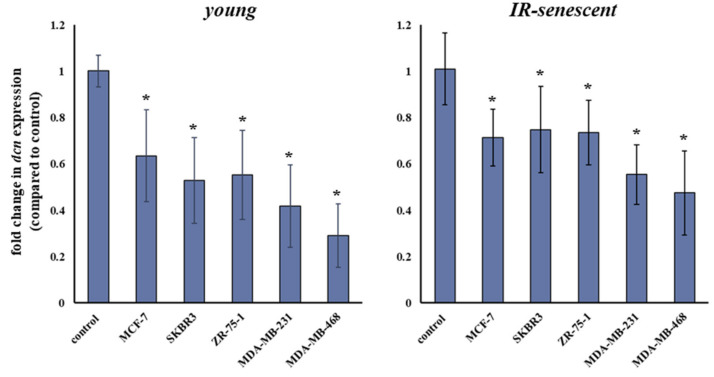
Human breast cancer cells decrease decorin expression in human stromal fibroblasts via a paracrine effect. Young and ionizing radiation (IR)-induced senescent human breast stromal fibroblasts were serum starved for 24 h and then incubated with media conditioned by the designated human breast cancer cell lines (MCF-7, SKBR3, ZR-75-1, MDA-MB-231 and MDA-MB-468) for another 24 h. RNA was extracted and decorin expression was assessed by real-time PCR after first-strand cDNA synthesis. Relative decorin mRNA levels to the respective untreated control with glyceraldehyde-3-phosphate dehydrogenase (GAPDH) serving as the reference gene are presented as means ± standard deviations of five independent experiments. Statistically significant differences from the respective untreated control are depicted by asterisks (*) (Student’s t test, *p* < 0.05).

As mentioned above, secreted growth factors by breast cancer cells have been shown by our laboratory to affect the expression of the proteoglycan syndecan-1 in human breast stromal fibroblasts [19]. This paracrine action was found to be dependent on the aggressiveness of the breast cancer cell lines investigated, with the triple-negative MDA-MB-231 cells resulting in syndecan-1 overexpression, while the low-invasive MCF-7 cells had no effect. The main growth factor participating in syndecan-1 overexpression has been demonstrated to be TGF-β [19]. In this direction and having already shown above that all exogenously supplied growth factors lead to decorin down-regulation, we assessed the paracrine effect of several breast cancer cell lines on decorin expression in young and senescent human breast stromal fibroblasts. We tested five breast cancer cell lines with different molecular profiles, i.e., MCF-7 (ER^+^/PR^+^), SKBR3 (HER2^+^), ZR-75-1 (ER^+^), MDA-MB-231 and MDA-MB-468 (both ER^−^/PR^−^/HER2^−^). As shown in Figure 7, all breast cancer cell lines resulted in the decrease in decorin expression in both young and senescent human breast stromal fibroblasts.

### 3.8. Ionizing Radiation Is Growth-Inhibitory for Breast Cancer Cells, But Does Not Alter Their Restraining Paracrine Effect on Human Breast Stromal Fibroblasts’ Decorin Expression

**Figure 8:**
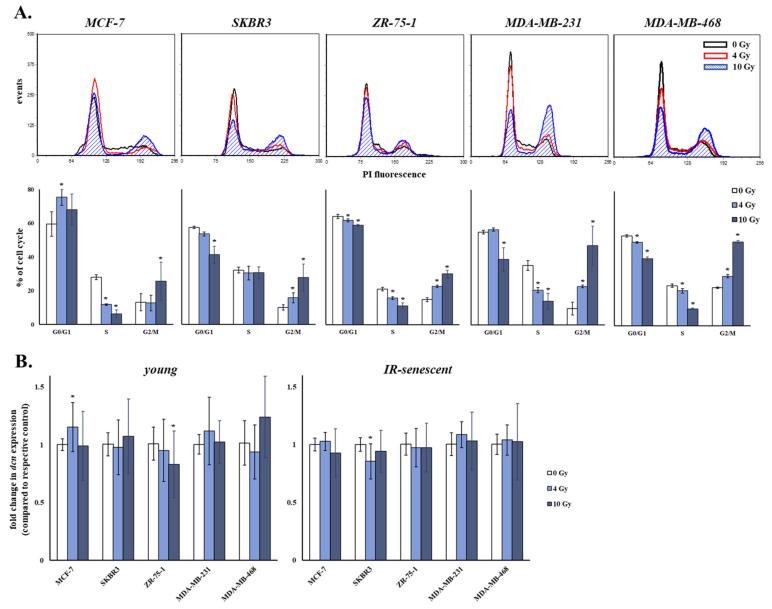
Ionizing radiation causes a cell cycle arrest in human breast cancer cells, which does not affect their paracrine effect on decorin expression of human breast stromal fibroblasts. (**A**) Breast cancer cells were exposed to 4 and 10 Gy of γ-irradiation. Cells were harvested after 24 h, stained with propidium iodide (PI) and analyzed by flow cytometry. Representative histogram plots are presented here, while the numerical values are the means ± standard deviations of three experiments. Asterisks (*) represent statistically significant differences in comparison to 0 Gy (Student’s *t* test, *p* < 0.05). (**B**) Young and ionizing radiation (IR)-induced senescent human breast stromal fibroblasts were serum starved for 24 h and then incubated with media conditioned by the designated human breast cancer cell lines (MCF-7, SKBR3, ZR-75-1, MDA-MB-231 and MDA-MB-468) treated with 0, 4 or 10 Gy for another 24 h. RNA was extracted and decorin expression was assessed by RT-qPCR. Relative decorin (dcn) mRNA levels to the respective control treated with the conditioned medium of 0 Gy are demonstrated as mean values ± standard deviations of three independent experiments. Glyceraldehyde-3-phosphate dehydrogenase (GAPDH) was used as the reference gene. Statistically significant differences from the respective control treated with the conditioned medium of 0 Gy are depicted by asterisks (*) (Student’s *t* test, *p* < 0.05).

During radiotherapy breast cancer cells are exposed to γ-irradiation in an attempt to eliminate tumor size by selectively killing or inhibiting the growth of cancer cells. Cancer cells exposed to ionizing radiation, but not killed, may adopt an altered phenotype and secretome that could differentially interact with the stroma. For that reason, we assessed the effect of two γ-irradiation doses on the cell cycle progression of breast cancer cells. In particular, we used a typical therapeutic dose (i.e., 4 Gy) and a dose of 10 Gy, frequently used for the inhibition of cancer cells’ growth. Both doses of γ-irradiation led to the cell cycle arrest of all five breast cancer cell lines investigated 24 h post-irradiation (Figure 8A). All breast cancer cell lines activated the G2/M checkpoint, while MCF-7 cells (harboring a wild-type p53 gene) activated the G1 cell cycle checkpoint, as well. The percentage of cells accumulating in the S phase was in most cases decreased, indicative also of the lower proliferation rate of breast cancer cells as a response to ionizing radiation.

We then assessed if breast cancer cells treated with ionizing radiation had an altered paracrine effect on the expression of decorin of young and senescent human breast stromal fibroblasts. As shown in Figure 8B, decorin expression levels of both young and senescent human breast stromal fibroblasts exposed to the conditioned medium of breast cancer cells untreated or treated with 4 and 10 Gy of ionizing radiation were similar.

### 3.9. Inhibition of bFGF and VEGF Annuls the MDA-MB-231 Conditioned Medium-Induced Decorin Down-Regulation in Young and Senescent Human Breast Stromal Fibroblasts

**Figure 9:**
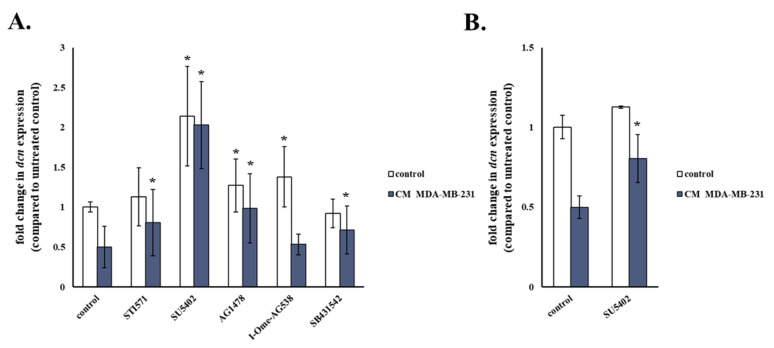
bFGF and VEGF inhibition abrogates decorin down-regulation mediated in human breast stromal fibroblasts by the paracrine action of MDA-MB-231 cells. Young (**A**) and ionizing radiation-induced senescent (**B**) human breast stromal fibroblasts were serum starved for 24 h, pre-incubated for 1 h with the growth factor receptor inhibitors (STI571 for PDGFR (2 μΜ); SU5402 for FGFR and VEGFR (20 μΜ); AG1478 for EGFR (3 μΜ); I-OMe-AG538 for IGFIR (12 μΜ); SB431542 for TGFβR1 (10 μΜ)) and then exposed to 50% (*v*/*v*) medium conditioned by MDA-MB-231 cells (CM MDA-MB-231). RNA was extracted and decorin (dcn) expression was estimated by real-time PCR after first-strand cDNA synthesis. Glyceraldehyde-3-phosphate dehydrogenase (GAPDH) was used as the reference gene. Relative dcn expression of treated cells to that of the untreated control is presented as mean ± standard deviation of three experiments. Statistically significant differences in comparison to the respective untreated control are marked by asterisks (*) (Student’s t test, *p* < 0.05).

Since we showed earlier that paracrine growth factors regulate decorin expression in human breast stromal fibroblasts, we continued by assessing whether inhibition of those growth factors’ receptors abrogates the decreased expression of decorin stimulated by the interaction of stromal fibroblasts with one selected breast cancer cell line, the highly invasive MDA-MB-231. As shown in Figure 9, nearly all growth factor receptor inhibitors partly reversed the ability of MDA-MB-231 conditioned medium to decrease decorin expression, indicating the release of growth factors from these cells, as reported [19]. However, once again SU5402 not only had the most prominent effect, but also increased decorin expression above basal levels. These data are in agreement with the release of FGFs from MDA-MB-231 [50], able—as shown earlier—to control also VEGF expression but also probably indicate the establishment of an autocrine loop by the other growth factors via bFGF.

## 4. Discussion

The active participation of the stroma in cancer development and tumor progression is now well established [10,13,14,15]. More specifically, stromal fibroblasts (the main stromal cell type charged with the production of ECM components, ECM proteolytic enzymes and chemokines) may create—depending on the received extracellular stimuli—either a more prohibitive or a more permissive environment for tumor growth [51,52,53,54].

The mammary gland is a very complex tissue and cell fate and differentiation of the epithelial parenchyma are determined by the highly regulated signals originating from the stroma even during normal development [55]. Furthermore, in breast cancers, the tumor-associated stroma constitutes a critical regulator of malignant conversion and progression [55]. Indeed, it has been reported that breast tumors rarely develop in the absence of a specialized stroma [55,56,57].

Radiotherapy has been undoubtedly proven to be an efficient remedy for breast cancer [58]. However, the genotoxic ionizing radiation provokes, in parallel, rapid, persistent, and comprehensive modifications in the mammary microenvironment, as evidenced by the altered ECM composition and growth factor activities [59], which could be largely attributed to the changed secretome of, the predominant cell type in this milieu, stromal fibroblasts. Normal human fibroblasts have been reported to become prematurely senescent as a response to ionizing radiation [18,25,26]. Originally, cellular senescence is adopted by the cells inhibiting uncontrolled proliferation and carcinogenesis [21,60,61]; the forfeit of this escape from cancer mechanism though is that accumulating senescent cells may in due course subserve neoplastic cells and prove beneficial for tumor expansion [62]. This is primarily driven by the phenotypic shift of senescent cells towards cells-producers of pro-inflammatory metabolites and catabolic molecules, the so-called SASP [4,5,21,24]. Nevertheless, although the implication of soluble SASP constituents and proteolytic enzymes in tissue remodeling has been thoroughly investigated [63,64], the study of the role of insoluble SASP factors, such as ECM components, has lagged behind. We have shown in a previous work of our laboratory that—similarly to fibroblasts of other origin—ionizing radiation induces premature senescence of human breast stromal fibroblasts, as well [19]. Along with a collagenolytic phenotype (decreased type I collagen synthesis and increased MMP activity), these senescent human breast stromal fibroblasts display overexpression of the proteoglycan syndecan-1 that represents a poor prognostic factor for tumor development when expressed in the stroma. Syndecan-1 overexpression is regulated by an autocrine loop orchestrated by TGF-β through the activation of the Smad pathway and the transcription factor Sp1 and is further enhanced by the highly invasive human breast cancer cells MDA-MB-231, both in young and senescent fibroblasts, via a paracrine action of TGF-β [19].

Members of the SLRP family of proteoglycans have been detected in breast tissues, with decorin and lumican being the main representatives, followed by biglycan and fibromodulin [65,66,67]. As decorin is the prototype SLRP member, here we assessed the expression profile of this ECM proteoglycan in ionizing radiation-induced senescent human breast stromal fibroblasts. Decorin has been reported to be significantly expressed in both healthy and various malignant conditions of human breast tissue but presents a stroma-exclusive distribution even in breast cancers, as human breast malignant cells have been shown to hardly express and synthesize decorin [65,68]. Given its ability to regulate stromal integrity by affecting fibrillary collagen cross-linking and growth factor signaling, increased decorin expression has been connected with regions of high mammographic density, corresponding to great collagen concentration and the extent of fibrosis [69]. Decorin is generally considered an effective antitumor molecule [28,29] and its genetic ablation enhances tumorigenesis by abetting the lack of known oncosuppressive genes (such as p53) [70,71] or by decreasing the levels of the cyclin-dependent kinase inhibitor p21^WAF1^ [72]. Due to its broad binding repertoire, including ECM constituents, multiple growth factors and receptor tyrosine kinases, decorin holds a multifaceted role in cancer and has been considered “a guardian from the matrix”, as it attenuates paracrine signals that would otherwise facilitate malignant transformation [35,73]. In addition, it has been demonstrated to perturb tumor angiogenesis [74], to elicit apoptosis in a squamous cell carcinoma model [75], to induce mitophagy [76] and to confine the metastatic spreading of breast carcinoma cells through the down-regulation of EGFR activity [30,77]. In accordance, low decorin levels have been associated with large tumor size, poorer survival and greater risk of early recurrence (especially when combined with high EGFR expression) in lymph node-negative invasive breast carcinomas [78].

We found that the typical molecular traits of ionizing radiation-induced senescent human stromal breast fibroblasts (i.e., the increased lysosomal function, as evidenced by the enhanced β galactosidase activity and lipofuscin accumulation, and the diminished proliferative potential, as a result of p16^INK4a^ and p21^WAF1^ up-regulation) were accompanied by the down-regulation of decorin. This senescence-associated decrease in decorin expression was observed at the transcriptional and protein level. Most importantly, decorin incorporation within the pericellular ECM deposited by cultures of senescent human breast stromal fibroblasts was also found to be eliminated in comparison to that detected in the autologous ECM produced by young cultures. We have previously reported that decorin levels are decreased in oxidative stress-induced prematurely senescent human intervertebral disc cells [42], while a reduced size of the decorin GAG chain has been shown in aged, compared to young, human skin [79]. Added to the declined collagen biosynthesis, the enhanced MMP activity and the plasminogen activator inhibitor-1 (PA-1), FGF and syndecan-1 overexpression reported previously to characterize senescent breast stromal fibroblasts [19,80,81,82], decorin down-regulation comes to reinforce the already tumor-promoting phenotype of these cells. It should be noted that lumican has been also shown to be down-regulated in human skin fibroblasts during ageing [83] and in senescent lung fibroblasts [84], while the expression of both decorin and lumican in the ECM can in turn affect tissue architecture by regulating type I collagen fibril structure [85] or by directly interacting with the catalytic domain of MMPs [86].

The reduced expression of decorin was demonstrated to be a long-term effect, since it seemed to augment along with the number of subcultures post-irradiation, while no change in decorin transcriptional levels was observed within the first 24 h after exposure of the cells to ionizing radiation. In favor of our findings, exposure of mice to acute low γ-irradiation doses induced significant down-regulation of decorin protein levels in the neonatal mouse heart seven months later [87], while decorin was suppressed in healthy mouse tissues 30 days post-exposure to high linear energy transfer radiation [88].

We then assessed the effect of selected paracrine growth factors known to be secreted by breast cancer cells, to be binding partners of decorin and to have an already shown paracrine action on syndecan-1 (i.e., PDGF, bFGF, EGF, IGF-I and TGF-β) [19,35,89,90] on the transcriptional regulation of decorin in human breast stromal fibroblasts. We found that all growth factors tested resulted in decorin down-regulation. In agreement with our data, decorin mRNA levels have been reported to be down-regulated in smooth muscle cells after treatment with TGF-β1 [91] and in an osteoblast precursor cell line after treatment with TGF-β1 or bone morphogenetic protein-2 (BMP-2) [92,93]. The addition of exogenous TGF-β1 decreased decorin expression in satellite cells during differentiation [94]. Decorin production was down-regulated in human skin fibroblasts treated with TGF-β2, which was reversed by co-treatment with dexamethasone [95]. Moreover, TGF-β has been reported to down-regulate decorin gene expression in an additive manner with tumor necrosis factor-α (TNF-α) in normal diploid fibroblasts [96]. TGF-β1 and bFGF were the strongest suppressors of decorin expression at the protein level in human colon cancer associated fibroblasts (CAFs) [97]. On the other hand, decorin biosynthesis was not significantly affected by PDGF or TGF-β1 treatment in monkey arterial smooth muscle cells [98] and incubation with bFGF led to the up-regulation of decorin mRNA levels in normal and keloid dermal fibroblasts [99], discords to our findings that could be attributed to tissue- and/or species-specific variations. In agreement to the above, inhibition of several growth factors’ receptors resulted in the up-regulation of decorin expression in both young and senescent human breast stromal fibroblasts. Notably, SU5402 (that blocks both FGF and VEGF) had the most prominent effect. Even more, pre-incubation of human breast stroma fibroblasts with SU5402 not only elevated basal decorin mRNA levels, but also annulled the decreased transcription of the molecule induced by the other growth factors, suggesting that all growth factors tested may act via bFGF and/or VEGF.

Given the dual inhibitory effect of SU5402 on both VEGFR and FGFR, we attempted to uncouple the action of the two growth factors by using a VEGFR- and a FGFR-specific inhibitor (i.e., SU5416 and AZD4547, respectively) in order to elucidate the mechanism of decorin down-regulation in young and senescent human breast stromal fibroblasts. Based on our results, both VEGF and bFGF repressed decorin expression in young cells, while VEGF appears to be the key player in decorin down-regulation in senescent cells. The catabolic/anti-anabolic role of bFGF and its inhibitory effect on proteoglycan production and especially on decorin expression has been previously reported for articular chondrocytes [100]. In addition, lower decorin expression has been described for osteoblasts carrying the activating FGFR2 mutations C342Y and S252W, found in the Crouzon and Apert syndrome, respectively [101].

It has been demonstrated that decorin requires the VEGFR2 kinase for successful autophagy via paternally expressed gene 3 (PEG3) [102]. Here, we showed that VEGF itself may exert an inhibitory action on decorin transcriptional regulation in young and senescent human breast stromal fibroblasts. Interestingly, even though the specific VEGFR inhibitor SU5416 enhanced decorin expression, exogenous administration of VEGF to human breast stromal fibroblasts did not result in decorin down-regulation. This finding pinpointed towards an intracellular action of VEGF, given that VEGF intracrine functions have been described, especially in non-endothelial cells [103]. Indeed, FGFR and/or VEGFR inhibition by SU5402, AZD4547 and SU5416 resulted in decreased VEGF mRNA levels in human breast stromal fibroblasts. SU5416—besides a specific VEGFR inhibitor—has been reported to inhibit VEGF mRNA expression in ovarian cancer cells through the PI3K/AKT/p70S6K1 signaling pathway [104]. On the other hand, VEGF decreased expression after FGFR inhibition (by SU5402 and AZD4547) suggested that VEGF is controlled by bFGF. In favor of this, supply of bFGF to human breast stromal fibroblasts led to VEGF overexpression. This is in agreement with the previously reported FGF activation of VEGF expression in mouse stromal embryonic fibroblasts [105]. Moreover, increased expression and secretion of VEGF has been reported to occur in senescent primary human fibroblasts [106].

Recently, it has been shown that autophagy activation induces decorin at the mRNA and protein level in vitro and in vivo, a process regulated at the transcriptional level via the inhibition of the canonical mTOR pathway [49]. In agreement to this, treatment with the mTOR inhibitor rapamycin led to decorin up-regulation in both young and senescent human breast stromal fibroblasts, while the autophagy inhibitor chloroquine resulted in lower decorin mRNA levels, providing evidence of an autophagy-mediated regulation of decorin expression in our cell model, as well. From this point of view, the senescence-associated decorin down-regulation described here for human stromal fibroblasts could be also linked to changes in the autophagic flux, since senescence and autophagy are interrelated cellular responses [107,108]. It is worth noting that inhibition of autophagy in senescent human breast stromal fibroblasts resulted in a significant increase in VEGF mRNA levels, which, as we showed here, seems to be an essential growth factor mediating decorin down-regulation in these cells.

As mentioned above, in the cancerous and pericancerous tissue, neoplastic and stromal cells inevitably interact due to their spatial contiguity and any anticancer treatment (such as ionizing radiation) has an effect on both malignant and normal stromal cells. We showed that exposure of human breast stromal fibroblasts to the soluble factors secreted by five human breast cancer cell lines of distinct molecular profile and degree of invasiveness (i.e., MCF-7, SKBR3, ZR-75-1, MDA-MB-231 and MDA-MB-468 [109,110]) resulted in decorin down-regulation in young and senescent cells. This is in agreement with previous bibliographic data showing decreased decorin protein expression in human CAFs treated with SKBR3- and MDA-MB-231-derived secretomes [97] and decreased decorin production in human LX2 stellate cells upon exposure to the conditioned media of different hepatoma cell lines (Hep3B, HLE, HepG2, and HuH7) [111]. In order to elucidate if γ-irradiation used in the clinical practice to inhibit breast tumors has any effect on the phenotype and secretome of the resistant or escaping apoptosis breast cancer cells, we exposed them to 4 and 10 Gy of ionizing radiation, monitored their cycle progression and collected their conditioned culture medium. Exposure to γ-irradiation blocked, as expected, the progression of the cell cycle in all breast cancer cell lines tested at the G2/M phase. MCF-7 cells were found to also activate the G1 checkpoint, since they carry a functional p53 gene [112,113]. Our data coincide with previously published information on the growth inhibitory action of ionizing radiation on breast cancer cell lines [114,115,116]. Despite provoking their cell cycle arrest, ionizing radiation did not seem to alter the secretome of breast cancer cells, at least regarding its ability to restrain decorin expression in human breast stromal fibroblasts.

Having shown earlier the implication in decorin down-regulation of growth factors produced in an autocrine manner or exogenously supplied to human breast stromal fibroblasts, we pre-incubated the cells with growth factor receptor inhibitors before exposing them to the conditioned medium of breast cancer cells. The breast cancer cell line MDA-MB-231 was selected based on its high grade of aggressiveness. We found that inhibition of FGF and VEGF reversed the MDA-MB-231 conditioned medium-induced decorin down-regulation in young and senescent human breast fibroblasts, indicating that FGF is released—amongst other soluble factors—by MDA-MB-231 breast cancer cells and can act in a paracrine manner on breast stromal cells directly and/or indirectly by enhancing stromal VEGF overexpression, ultimately inducing the decrease in their decorin expression. Interestingly, both FGF and VEGF are potent pro-angiogenic factors [117] and thus—having in mind the anti-angiogenic role of decorin—this FGF- and VEGF-induced decorin down-regulation may further promote vascularization favoring tumor growth.

## 5. Conclusions

**Figure 10:**
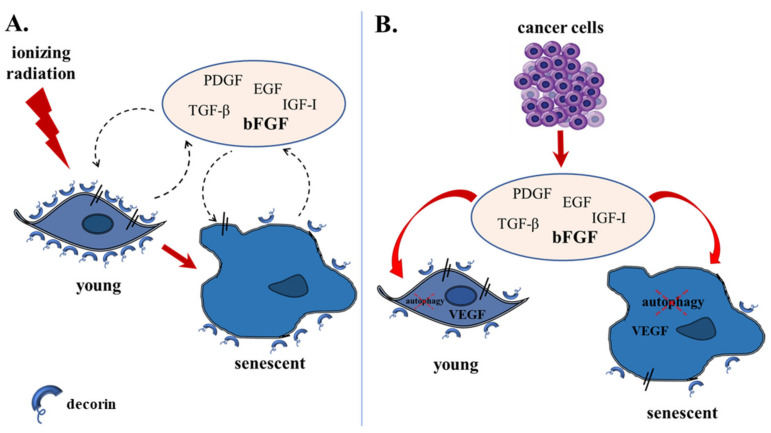
A model presenting decorin down-regulation in human breast stromal fibroblasts as a consequence of ionizing irradiation-mediated premature senescence and the interaction with invasive breast cancer cells. (**A**) Ionizing radiation leads young human breast stromal fibroblasts to premature senescence. (**B**) Highly invasive breast cancer cells induce decorin down-regulation in young breast stromal fibroblasts and an extra decline in decorin expression in senescent breast stromal fibroblasts. Decorin down-regulation is associated with autophagy inhibition and is mediated by an autocrine loop, as well as by the paracrine action of cancer cells, with bFGF secreted in the extracellular space being the key player, controlling VEGF expression and possible intracrine action.

In conclusion, here we showed that ionizing radiation-induced human breast stromal fibroblasts are characterized by the down-regulation of the SLRP decorin, resulting in its lower mRNA and protein levels and its reduced deposition in the pericellular ECM. Decorin down-regulation is mediated by PDGF, EGF, IGF-I, TGF-β and principally by bFGF secreted by the stromal cells themselves acting in an autocrine manner, as well as that released by the neighboring aggressive breast cancer cells establishing a paracrine effect. bFGF was shown to act—at least partly—via VEGF overexpression and possible intracrine action. VEGF overexpression and decorin down-regulation were reversed by autophagy activation (Figure 10). As part of its role as a “guardian from the matrix” [35], one of the main actions of decorin is to bind growth factors, thus interfering with their effects.

Decorin’s biological activity is rather complex as it regulates multiple processes in the ECM and in the tumor cells. Its antitumorigenic role though is unequivocal, having rendered decorin expression in the cancer-associated stroma a molecular predictor for the prognosis of the disease. Specifically for breast cancer patients, low levels of decorin in the tumor microenvironment are associated with a more aggressive disease phenotype [118,119,120]. The decreased decorin expression in prematurely senescent human breast stromal fibroblasts induced by ionizing radiation (a typical treatment for breast cancer) shown here fulfills the already tumor-promoting phenotype of these cells, which have been shown to display increased MMP activity and syndecan-1 up-regulation (also considered a poor prognostic factor for breast cancer progression) [19]. Since decorin is a known-negative TGF-β regulator [121], decreased expression of decorin in senescent breast stromal cells may result in the release of TGF-β and in further TGF-β-mediated syndecan-1 up-regulation and further TGF-β-induced decorin down-regulation. Senescent cells with the afore-mentioned phenotypic traits inevitably arise as a side-effect of genotoxic anticancer treatments. It becomes thus evident that counteracting senescent cells in the stroma could represent an additional molecular target along with the efficient elimination of neoplastic cells in generalized anticancer treatment regimes.

## Figures and Tables

**Table 1:** Primer sequences (5′ → 3′).

Target Gene	Forward Primer	Reverse Primer
*p16^INK4a^*	TAGTTACGGTCGGAGGCCGAT	GCACGGGTCGGGTGAGAG
*p21^WAF1^*	CTGGAGACTCTCAGGGTCGAA	CCAGGACTGCAGGGTTCCT
*dcn*	CCTGATGACCGCGACTTCGAG	TTTGGCACTTTGTCCAGACCC
*vegf*	CCTCCGAAACCATGAACTTT	TTCTTTGGTCTGCATTCACATT
*gapdh*	GAGTCCACTGGCGTCTTC	GCATTGCTGATGATCTTGAGG

## Data Availability

The data presented in this study are available in this article.

## Supplementary Materials

The following are available online at https://www.mdpi.com/article/10.3390/cancers13081987/s1, Figure S1: Uncropped blots and densitometric analyses of Western blot experiments.
