# Supplementary figures and images for: Down-Regulation of the Proteoglycan Decorin Fills in the Tumor-Promoting Phenotype of Ionizing Radiation-Induced Senescent Human Breast Stromal Fibroblasts
Source: Cancers (Basel). 2021 Apr 20;13(8):1987. doi: 10.3390/cancers13081987 (PMC8074608; doi:10.3390/cancers13081987)

# Original images of blots and densitometric analysis

## Figure 1C

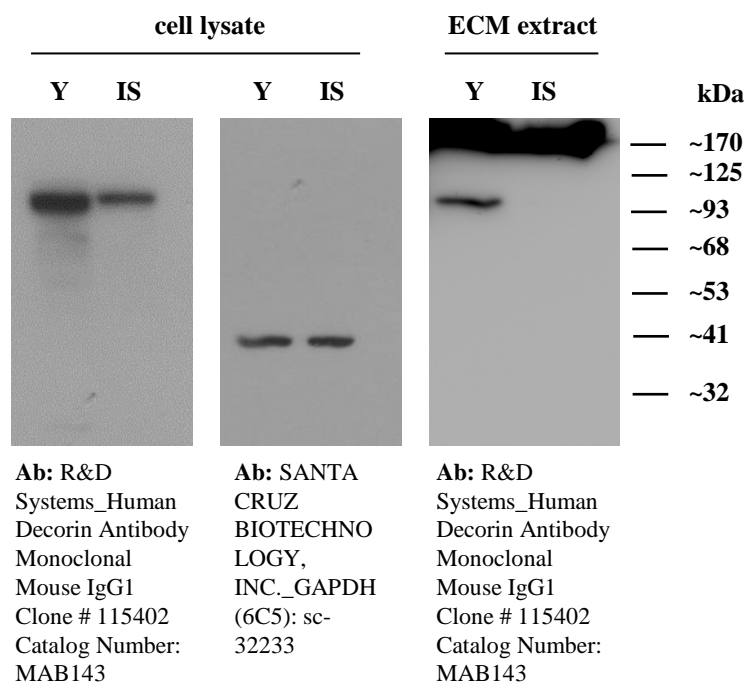

### Densitometric analysis

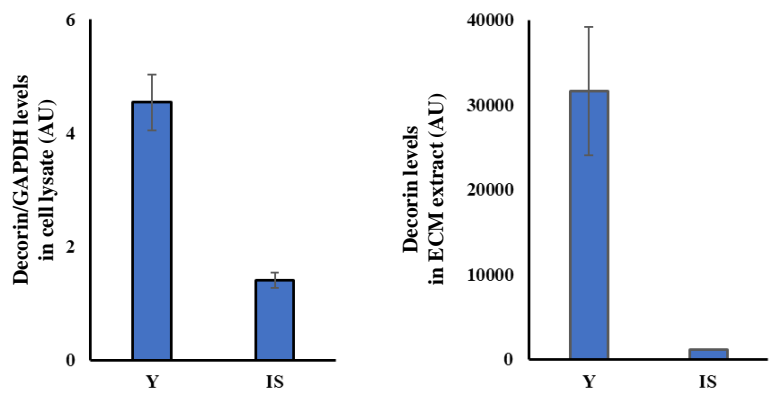

Supplement: Supplementary file 1 [file cancers-13-01987-s001.zip › cancers-1193951-supplementary.pdf]
